# Global testing of shifts in metabolic phenotype

**DOI:** 10.1007/s11306-018-1435-8

**Published:** 2018-10-04

**Authors:** Parastoo Fazelzadeh, Huub C. J. Hoefsloot, Thomas Hankemeier, Jasper Most, Sander Kersten, Ellen E. Blaak, Mark Boekschoten, John van Duynhoven

**Affiliations:** 1 Nutrition, Metabolism and Genomics Group, Division of Human Nutrition, Wageningen University, Wageningen, The Netherlands; 2 Swammerdam Institute of Life Sciences, University of Amsterdam, P.O. Box 94215, 1090 GE Amsterdam, The Netherlands; 3 Division for Analytical Biosciences, Leiden University, Leiden, The Netherlands; 4 Department of Human Biology, NUTRIM School of Nutrition and Translational Research in Metabolism, Maastricht University, Maastricht, The Netherlands; 5 Laboratory of Biophysics, Wageningen University, Wageningen, The Netherlands; 6 Top Institute Food and Nutrition, Wageningen, The Netherlands; 7 Netherlands Metabolomics Centre, Leiden, The Netherlands; 8 Unilever R&D, Vlaardingen, The Netherlands

**Keywords:** Goeman’s global test, Metabolic pathways, Phenotype shifts

## Abstract

**Introduction:**

Current metabolomics approaches to unravel impact of diet- or lifestyle induced phenotype variation and shifts predominantly deploy univariate or multivariate approaches, with a posteriori interpretation at pathway level. This however often provides only a fragmented view on the involved metabolic pathways.

**Objectives:**

To demonstrate the feasibility of using Goeman’s global test (GGT) for assessment of variation and shifts in metabolic phenotype at the level of a priori defined pathways.

**Methods:**

Two intervention studies with identified phenotype variations and shifts were examined. In a weight loss (WL) intervention study obese subjects received a mixed meal challenge before and after WL. In a polyphenol (PP) intervention study obese subjects received a high fat mixed meal challenge (61E% fat) before and after a PP intervention. Plasma samples were obtained at fasting and during the postprandial response. Besides WL- and PP-induced phenotype shifts, also correlation of plasma metabolome with phenotype descriptors was assessed at pathway level. The plasma metabolome covered organic acids, amino acids, biogenic amines, acylcarnitines and oxylipins.

**Results:**

For the population of the WL study, GGT revealed that HOMA correlated with the fasting levels of the TCA cycle, BCAA catabolism, the lactate, arginine–proline and phenylalanine–tyrosine pathways. For the population of the PP study, HOMA correlated with fasting metabolite levels of TCA cycle, fatty acid oxidation and phenylalanine–tyrosine pathways. These correlations were more pronounced for metabolic pathways in the fasting state, than during the postprandial response. The effect of the WL and PP intervention on a priori defined metabolic pathways, and correlation of pathways with insulin sensitivity as described by HOMA was in line with previous studies.

**Conclusion:**

GGT confirmed earlier biological findings in a hypothesis led approach. A main advantage of GGT is that it provides a direct view on involvement of a priori defined pathways in phenotype shifts.

**Electronic supplementary material:**

The online version of this article (10.1007/s11306-018-1435-8) contains supplementary material, which is available to authorized users.

## Introduction

Health is maintained by well-orchestrated interactions between physiological processes. These processes have to function in a changing environment and thus they collectively strive to maintain homeostasis by continuous adaptations. The ability to adapt to stressors such as diet and exercise has been coined as phenotypical flexibility (PF) and has been proposed as a measure for health (van Ommen et al. 2014; Huber et al. 2011). PF has been brought forward as a broad concept (van Ommen et al. 2014), but comprises the well-established concept of metabolic flexibility (MF), which is the efficiency of the postprandial switch between fasting lipid catabolism to postprandial carbohydrate anabolism (Corpeleijn et al. 2009). Several studies have claimed that phenotypical flexibility reflects the capacity to adapt to the new situation and this provides a better indication of health and disease risk comparing to fasting measure (Elliott et al. 2007; Vis et al. 2015; Fiamoncini et al. 2018). Challenge tests have been put forward to measure the phenotypic flexibility of a biological system by recording how well the system is able to undo a perturbation bringing the system back to steady state.

In earlier studies univariate statistics was used to find differences between metabolic phenotype, at the level of baseline metabolism or in the response to a challenge (van Ommen et al. 2014; Vis et al. 2015; Fiamoncini et al. 2018). This approach is compromised by the multiple testing problem, and also cannot unambiguously establish whether a phenotype shift is better reflected in a shift in fasting metabolism or in an altered response to a dietary challenge. In this work we will explore an approach where we exploit prior knowledge on involvement of metabolic pathways in shifts in fasting metabolism or altered postprandial responses. In order to exploit this prior knowledge we will use Goeman’s global test, which is a robust test to identify whether metabolites that are connected in a pathway collectively respond to a change in conditions (Hendrickx et al. 2012). In the metabolomics field, predefined groups of pathways or functional modules can be used (Kanehisa and Goto 2000; Kanehisa et al. 2006, 2010).

**Fig. 1:**
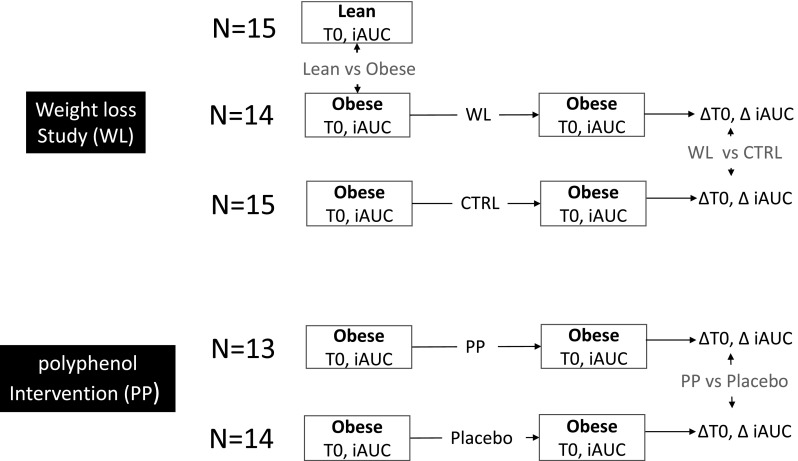
Schematic overview of Goeman’s global testing of differences in pathways between different phenotype groups (lean, obese) and due to WL and PP interventions. In the WL and PP intervention studies respectively also control (CTRL) and placebo groups were included. For all groups plasma was sampled at fasting state (T0) and during the postprandial response to a meal challenge. The postprandial response was captured in the incremental area under the curve (iAUC). The effect of the WL, CRTL, PP and placebo interventions was summarized in ΔT0 and ΔiAUC. The vertical arrows indicate application of Goeman’s test (indicated in red) to comparison of lean versus obese (based on T0 and iAUC) and on comparison of WL versus CRTL and PP versus placebo groups (based on ΔT0 and ΔiAUC)

We will explore this approach in two studies, schematically depicted in Fig. 1, where mixed-meal challenges were carried out to assess the efficacy of two types of dietary interventions. The first study examined whether a mixed meal challenge response could provide a readout for a shift in phenotypical flexibility upon weight loss (WL) in obese male subjects. This study showed a significant improvement in insulin sensitivity after WL (Joris et al. 2016) and thus provides a relevant case for testing our approach. In the second study, the effect of long term polyphenol (PP) consumption on phenotypical flexibility was also assessed by means of a mixed meal challenge. The PP intervention did not have an effect on insulin sensitivity and MF but increased fasting level and postprandial fat oxidation as compared to placebo (Most et al. 2016). In both studies the responses of amino acids and acylcarnitines were measured, as well as metabolites related to the TCA cycle. This set of metabolites was selected based on previous studies that most convincingly related them with the ability to effectively switch from lipid to carbohydrate metabolism in the postprandial phase, i.e. MF.

We explored Goeman’s global testing approach (Hendrickx et al. 2012; Goeman et al. 2004) to assess the effect of the WL and PP intervention on both fasting (at T0) and mixed meal challenge responses. Originally the Goeman test was developed for gene-expression data. This test is on predefined groups of genes thereby diminishing the multiple testing problems associated with testing all genes separately. If a treatment–control study is considered the Goeman test will tell how well the predefined genes can predict the membership of the treatment or control group. Classically logistic regression is the tool used for the prediction. The significance of the result is established by a permutation test. In this test the membership of the two groups, either treated or control, is randomized and this performance is compared to that of the original data. If now instead of gene-expression data, metabolomics data is used exactly the same statistical procedure can be followed (Hendrickx et al. 2012). Furthermore, we will use this approach to establish correlations between phenotypical parameters (insulin sensitivity and MF) and metabolic pathways.

## Materials and methods

### Subject characteristics

#### Weight loss intervention study

29 abdominally overweight/obese men (BMI = 30.0 ± 0.5 kg/m^2^ for WL and 30.7 ± 0.7 kg/m^2^ for control groups) participated in the study. None of the subjects were diagnosed with clinical diseases. Subjects characteristics can be found in Supplementary Table S1-a.

#### Polyphenol intervention study

38 overweight and obese subjects (BMI = 28.5 ± 0.8 for placebo and BMI = 29.6 ± 0.8 kg/m^2^ for PP intervention groups) participated in this study, 28 subjects that provided complete data were randomly selected for metabolomics analysis. Characteristics of the subjects who completed the study are summarized in Supplementary Table S1-b.

### Study design

#### Weight loss intervention study

Lean subjects were only studied cross-sectional, and obese/overweight subjects before and after random assignment to a WL intervention of 8 weeks. Before and after the WL intervention (D1 and D2), all subjects underwent a mixed meal challenge test and blood samples were collected during 4 h. Subjects were randomly assigned to either a WL or control (CTRL) program for 8 weeks, see Fig. 1 for a schematic depiction. HOMA was calculated at both D1 and D2. Details on the design have been published (Joris et al. 2016).

#### Polyphenol intervention study

In this randomized, double-blind, placebo-controlled, parallel-intervention trial, subjects received either a PP supplement (epigallocatechin gallate and resveratrol; 282 and 80 mg/day, respectively) or a placebo (partly hydrolyzed microcrystalline cellulose-filled capsules) for a period of 12 weeks to assess effects of PP supplementation on tissue-specific insulin sensitivity (primary outcome) and plasma metabolic profile, fat oxidation, skeletal muscle oxidative capacity, and lipolysis (secondary outcomes). The supplementation period started the day after the last measurement in week 0 and was continued throughout measurements in week 12. In this study we use the data from the high-fat mixed meal (HFMM) challenges that were performed before supplementation was initiated, and after 12 weeks before supplementation was stopped, see Fig. 1 for a schematic depiction. In addition to HOMA, energy expenditure, respiratory quotient (RQ), fat and carbohydrate oxidation were measured by indirect calorimetry using the open-circuit ventilated hood system (Omnical; Maastricht University) and were calculated according to the formulas of Weir and Frayn, respectively. A hyperinsulinemic–euglycemic clamp with an isotope labelled glucose infusion tracer was performed to assess rate of disappearance (Rd, as measured for peripheral insulin sensitivity) and endogenous glucose production (% EGP a measure for hepatic insulin resistance). Full details of the study can be found elsewhere (Most et al. 2016).

### Sample collection

#### Weight loss intervention study

Subjects were asked not to perform any strenuous physical exercise or to consume alcohol and high-fat foods on the day before blood sampling. Blood samples were taken at fasting and after mixed meal consumption both before and after the WL intervention at six time points (fasting (T0) and 30, 60, 120, 180 and 240 min in the postprandial state). The standardized mixed meal consisted of two muffins and 300 ml 0% fat milk, which provided 1100 kcal: 56.6 g fat, 26.5 g protein and 121 g carbohydrate. Metabolic profiling was performed on all time points. Full details can be found in Joris et al. (2016).

#### Polyphenol intervention study

After inserting a cannula into the antecubital vein, substrate oxidation was measured for 30 min under fasting conditions (T0) and for 4 h after the ingestion of a liquid HFMM (625 kcal, 61% of energy from fat, 33% of energy from carbohydrate, 6% of energy from protein), which was consumed within 5 min at *t* = 0. Blood samples were taken under fasting (0 min) and postprandial (*t* = 30, 60, 90, 120, 150, 180, 210, and 240 min) conditions. Full details of the study can be found elsewhere (Most et al. 2016).

### Plasma metabolic profiling

Full details on analytical procedures can be found in Fazelzadeh et al. (2018). In short, amino acids and biogenic amines, organic acids and acylcarnitines were measured for both the WL and PP intervention studies, with a total 170 metabolites. Amino acids and biogenic amines in plasma were derivatized (Acc-TAG) and measured by a UPLC system which was interfaced to quadrupole mass spectrometer. Acylcarnitines in plasma were also measured by UPLC–MS, but without derivatization. Organic acids in plasma were measured by GC–MS, after oximation and silylation derivatization. Oxylipins were only analyzed for the WL study. First an SPE extraction was performed and subsequently a LC separation coupled to ESI on a triple quadrupole mass spectrometer. Oxylipins were detected in negative ion mode using dynamic SRM. Full details of these platforms have been described in earlier studies (Noga et al. 2012; van der Kloet et al. 2009; Mihaleva et al. 2014; Fazelzadeh et al. 2018). Serum metabolites were measured by NMR in a quantitative manner, full experimental details can be found in earlier work (Mihaleva et al. 2014). In short, serum samples were ultrafiltrated and automated quantum mechanical line shape fitting of ^1^H NMR spectra was performed using PERCH in order to obtain absolute metabolite concentrations.

### Statistical analysis

For pathway analysis, Goeman’s global test for metabolomics was applied to test groups of covariates (or metabolites) for association with a response variable using the global test R library (Hendrickx et al. 2012; Goeman et al. 2004). Nadir acylcarnitine values were defined as the lowest value achieved during the 4 h after the meal (Ramos-Roman et al. 2012). Δ nadir was calculated as difference between nadir and T0 values. All analyses were done using R (version 3.1.2). As a reference method we used Analysis of Variance (ANOVA) for between group comparisons. Linear mixed modelling was used to assess the fasting comparison and difference of response effect between groups. *P* < 0.05 was considered to be statistically significant. To account for multiple testing, local false discovery rates (lFDR) were calculated for each metabolite (Strimmer 2008a, b). The postprandial response was considered as incremental area under the curve (iAUC), taking the fasting measurement (t0) as a reference. The iAUC was calculated in two ways. First, by considering that the AUC comprises both negative AUC (AUC−) and positive AUC (AUC+) contributions, we refer to this value as iAUC. Secondly, we also calculated the positive iAUC where the absolute values contributing to the curve were summed up, we refer to these values as piAUC (Carstensen et al. 2003; Pellis et al. 2012). For both iAUC and piAUC we use the trapezoidal calculation method (Senn 1990).

## Results

### Univariate assessment of intervention effects and correlations with phenotype parameters

#### Weight loss intervention study

**Fig. 2:**
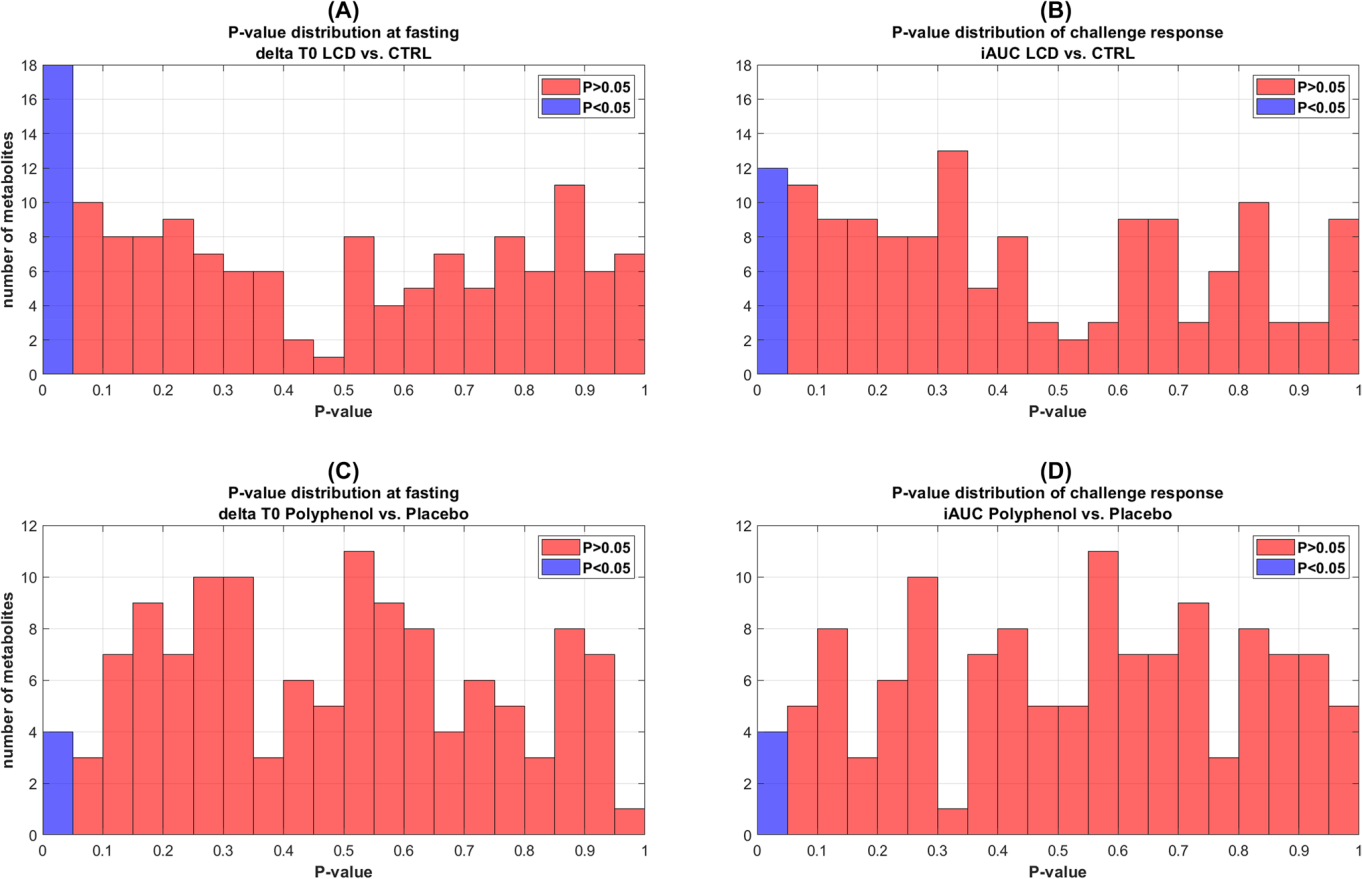
*P*-value distribution of effect of WL on fasting state values in obese subjects (ΔWL T0 vs. Δ CTRL T0). **a** Effect of WL on challenge response values in obese subjects (iAUC WL vs. iAUC CTRL). **b** Effect of PP intervention on fasting state values in obese subjects (Δ PP T0 vs. Δ placebo T0). **c** Effect of PP intervention on challenge response values in obese subjects (iAUC PP vs. iAUC placebo). **d** Metabolites with *P* < 0.05 were coloured green and otherwise red

Figure 2a, b shows the *P*-value distribution of values for the WL effect on respectively fasting metabolite levels and their postprandial response as expressed by iAUC. Comparing fasting and the challenge response between obese subjects that underwent WL or control interventions, we found that a range of metabolites are significantly different between two groups in either comparison. Figure 2a, b also show that there is a more pronounced effect at fasting as compared to the postprandial response. Metabolites that are significantly different between groups at fasting (Supplementary Table S2A) are not changed upon a mixed meal challenge (Supplementary Table S2B). Branched chain amino acids (BCAA) and amino acid derived acylcarnitines (AAAC) were among the most significantly different metabolites between groups in the fasting state.

#### Polyphenol intervention study

The P-value distribution of the PP intervention effect on metabolite fasting state and response (iAUC) values are presented in Fig. 2c, d, respectively. The P-value distribution shows that only a small number of metabolites are different between two groups either at fasting or in their postprandial response. This also in line with the small number of metabolites on which the PP intervention had a significant effect according to univariate testing (Supplementary Table S3A and B).

#### Correlation with phenotype parameters

For the WL study population a number of univariate correlations between metabolite levels and HOMA can be established (Fazelzadeh et al. 2018). The correlations between HOMA and branched chain amino acids, phenylalanine and tyrosine are in line with previous studies (Newgard 2012; Newgard et al. 2009). In addition, we could also find correlations between HOMA and C_2_/C_n_ acylcarnitine ratios, which were recently brought forward as putative read-outs for β-oxidation rate (Krug et al. 2012). In an earlier study, we also found correlation between HOMA and Δ nadir and nadir acylcarnitine levels, in particular for amino acid derived ones (Fazelzadeh et al. 2018). The Δ nadir and nadir acylcarnitine values can be considered as a single parameter summary of their postprandial response (Ramos-Roman et al. 2012) and the correlations with HOMA suggest they are related to fatty acid oxidation flux.

For the PP intervention study, we can observe a number of significant correlations between these phenotypical parameters with metabolites at fasting (Supplementary Table S4). These correlations however provide a scattered view on involvement of metabolic pathways. C_2_/2-methylbutyroylcanitine significantly correlated with HOMA (*P* = 0.02, ρ = − 0.31), but not with ΔRQ, or fat oxidation. ∆nadir and nadir acylcarnitine levels with phenotypic parameters (HOMA, ΔRQ, fat oxidation) did not show any significant correlations.

### Goeman’s global test for assessment of intervention effects on plasma metabolome

Metabolites from the TCA cycle and the lactate pathway were grouped according to the KEGG database. For amino acid and fatty acid derived acylcarnitines no pathway information has been entered in KEGG. Hence we grouped metabolites according to branched amino acid catabolism, comprising branched chain amino acids and derived acylcarnitines (AAACs, C3, C4, C4DC, C5). Fatty acid derived acylcarnitines (FAACs, C8–C18) were also grouped into one pathway. We also grouped phenylalanine and tyrosine, since both these amino acids have consistently been associated with insulin resistance (Newgard et al. 2009). Oxylipins derived from arachidonic acid were also grouped in a pathway. An overview of metabolites collected in pathways is given in Table 1. The result of Goeman’s global testing for assessment of lean versus obese differences and effect of WL in obese is presented in Table 2. Three pathways, including the TCA cycle and BCAA catabolism are different between obese and lean subjects at fasting. The combination of phenylalanine and tyrosine was also significantly different between lean and obese. The last two pathways were also different between obese subjects before and after a WL intervention. Regarding the postprandial metabolic response, the difference between obese and lean subjects was limited to lactate and the Arg–Pro pathway. We note that here we present effects for iAUC values, which account for positive and negative contributions. The effects are similar when piAUC values are considered (Supplementary Table S5). The Goeman’s global test did however not reveal significant (p > 0.05) WL induced differences in postprandial response in obese subjects. The effect of WL on the enzymatic oxidation pathway of arachidonic acid did not reach statistical significance (p < 0.09). The Goeman’s global testing approach was also deployed to reveal pathways that were affected by the PP intervention. No significant effect could however be observed for neither fasting metabolite levels nor the metabolic postprandial responses, irrespective of whether iAUC or piAUC values were used.

**Table 1:** Collection of metabolites in pathways for Goeman’s global testing, for more detail on the metabolites see Supplementary Table S6

Pathway	Number of metabolites in the group	Metabolites
TCA cycle	8	Citric acid, malic acid, 2-ketoglutaric acid, succinic acid, fumaric acid, pyroglutamic acid, *cis*-aconitic acid, pyruvate
BCAA catabolism	8	Val, lle, Leu, Amino acid acylcarinitnes (AAACs): C3, C4, C4DC, C5-Leu, C5-Ile
Fatty acid oxidation	16	Fatty acid acylcarnitines (FAACs): C6.0, C8.0, C8.1, C9.0, C10.0, C10.1, C12.0, C12.1, C14.0, C14.1, C14.2, C16.0, C16.1, C18.0, C18.1, C18.2
Lactate pathway	3	Lactate, pyruvate, glucose
Arg, Pro	6	Methionine, proline, ornithine, citrulline, 4-hydroxyproline, arginine
Phe, Tyr	2	Phenylalanine, tyrosine
Enzymatic oxidation of arachidonic acid	5	TXB2, PGE2, 12S.HHTrE, 5.HETE, 11.HETE

**Table 2:** Goeman’s global testing of differences in metabolic pathways (Table 1) between lean and obese volunteers and the effect of a WL intervention [compared to a control (CRT) intervention]

Pathways and number of metabolites		Fasting		Response	
		T0	∆T0	iAUC	∆iAUC
		Obese versus lean	WL versus CRTL	Obese versus lean	WL versus CRTL
TCA cycle	8	0.01	0.2	0.4	0.3
BCAA catabolism	7	0.02	0.02	0.5	0.5
Fatty acid oxidation	16	0.07	0.4	0.7	0.5
Lactate pathway	3	0.06	0.5	0.03	0.3
Arg, Pro	6	0.5	0.1	0.02	0.6
Phe, Tyr	2	0.01	0.03	0.3	0.3
Enzymatic oxidation of arachidonic acid	5	0.6	0.5	0.3	0.09

Differences were tested for fasting state values (T0) and postprandial response (iAUC)

### Goeman’s global test for assessment of correlations of plasma metabolome with phenotypical variables

The populations of the WL and PP intervention studies were well characterized with respect to insulin sensitivity (HOMA), MF (RQ) and fasting state fat oxidation (Supplementary Table S1a, b). HOMA was determined in both the WL and PP intervention studies, whereas RQ and fasting state fat oxidation were only determined in the PP study. We explored whether the Goeman’s global testing approach would provide a means to establish correlations between phenotype parameters and metabolic pathways. Goeman’s global test revealed that for the population of the WL intervention study HOMA correlated with the fasting levels of metabolites from the TCA cycle, BCAA catabolism, lactate–glucose, arginine–proline and phenylalanine–tyrosine pathways (Table 3). For this population the correlation between pathways and HOMA were more pronounced at fasting and less in the postprandial response as expressed by iAUC (Table 3) or piAUC (Supplementary Table S5).

**Table 3:** Goeman’s global test results for correlations between fasting state values, postprandial response (expressed as iAUC) and HOMA

Pathways and number of metabolites		Fasting		Postprandial response
		Weight loss intervention group	Polyphenol intervention group	Weight loss intervention group
TCA cycle	8	**0.01**	**0.02**	0.4
BCAA catabolism	7	**0.04**	0.4	0.6
Fatty acid oxidation	16	0.7	0.06	0.4
Lactate pathway	3	**0.05**	0.9	**0.02**
Arg, Pro	6	**0.04**	0.6	0.7
Phe, Tyr	2	**0.02**	**0.04**	0.5
Enzymatic oxidation of arachidonic acid	5	0.7	0.6	0.4

The Goeman’s global tests were performed separately for volunteers from the WL and PP intervention studies. No significant effects were observed of effect of PP intervention on postprandial response. Note that no arachidonic acid (AA) metabolites (oxylipins) were measured for the PP intervention study. P-values ≤ 0.05 are indicated in bold

For the PP study Goeman’s global test revealed that HOMA is correlated with TCA cycle (P = 0.02) and fatty acid oxidation pathways (P = 0.06) as observed in fasting levels (Table 3). For ΔRQ, fat oxidation, hepatic insulin resistance and peripheral insulin sensitivity no correlations could be established for any of the pathways when fasting levels were considered. Goeman’s test also found no correlations between any of the phenotypical parameters (HOMA, ΔRQ, fat oxidation, hepatic insulin resistance, peripheral insulin sensitivity) and postprandial responses (iAUC and piAUC) when these were grouped in pathways.

## Discussion

### Global testing of intervention effects on metabolic pathways

We applied Goeman’s global test to determine whether sets of metabolites that are connected within a pathway collectively respond to an intervention. Our aim was to examine whether a phenotypic flexibility can be defined as a shift in fasting metabolism or by the postprandial metabolic response. A study by Hendrickx et al. (2012) revealed that Goeman’s global test can be used to determine if the behaviour of a group of metabolites within the same pathway, is related to a specific outcome of interest. We applied the Goeman’s global test on two studies where volunteers respectively underwent a WL and PP intervention. The WL intervention caused a more pronounced shift in fasting levels (T0) rather than in postprandial response as is shown in *P* distribution plot in Fig. 2. For the WL intervention Goeman’s global tests indeed showed significant effects at pathway level at fasting (Table 2). Metabolites involved in the TCA cycle have significantly different pattern between obese and lean subjects at fasting, which is in line with other studies (Newgard et al. 2009). Moreover, we observed an effect of WL on BCAA catabolism, and the combination of Phe–Tyr, which is in line with previous observations of changes in metabolic profiles accompanying an improvement in insulin resistance (Newgard 2012; Newgard et al. 2009). In previous work (Fazelzadeh et al. 2018), we observed that the main metabolites for which the postprandial response was different before/after WL were oxylipins derived from arachidonic acid by enzymatic oxidation. Although the individual *P* values were significant for arachidonic acid derived oxylipins, when we test them collectively in Goeman’s test the effect of WL on the postprandial response of this pathway was only *P* = 0.09.

In the dietary PP intervention study, the *P* distribution plot indicated that only a small number of metabolites were affected in the fasting state (T0) or in their postprandial response. This was also reflected in the small number of metabolites that showed a significant effect according to univariate testing (Supplementary Table S3). Goeman’s global test indeed did not reveal any pathways that were significantly different before and after the PP intervention. This is in line with the relative small effect of the PP intervention on phenotypical parameters. Although the prolonged PP supplementation stimulated fat oxidation and increased mitochondrial capacity comparing to placebo, no significant effect on tissue-specific insulin sensitivity and MF in obese subjects was observed (Most et al. 2016).

### Goeman’s global testing of correlations between pathways and phenotype parameters

The observed correlations of HOMA with metabolites involved in TCA cycle, BCAA catabolism and lactate pathway can be explained by their positive correlation with insulin resistance (Newgard et al. 2009; Newgard 2012). However, Goeman’s global test showed that the changes in postprandial response are smaller than the change at fasting after the intervention (Table 3). In the population of the WL study, more pathways were significant than for the PP intervention study, in particular at fasting. As the HOMA range of obese subjects for of two studies is comparable (Supplementary Table S1a, b), the lack of pronounced effects in PP study might be due lack of power and/or confounding with other phenotype parameters.

## Conclusion

The application of Goeman’s global test to two intervention studies indicates that it can provide a direct view on involvement of a priori defined pathways in phenotype shifts. The effect of WL intervention on a priori defined metabolic pathways was consistent with previous studies, as well as correlation of pathways with insulin sensitivity as described by HOMA. Goeman’s global test, indicated that in the two intervention studies shifts in metabolic phenotype were more strongly reflected in pathways observed at baseline, than in their postprandial response. This confirms the biological interpretation of univariate tests performed in these studies.

## Electronic supplementary material

Below is the link to the electronic supplementary material.

Supplementary material 1 (DOCX 29 KB)

Supplementary material 2 (DOCX 13 KB)
