# Supplementary material for: Global testing of shifts in metabolic phenotype
Source: Metabolomics. 2018 Oct 4;14(10):139. doi: 10.1007/s11306-018-1435-8 (PMC6208751; doi:10.1007/s11306-018-1435-8)
Supplement: Supplementary file 1 — Supplementary material 1 (DOCX 29 KB) [file 11306_2018_1435_MOESM1_ESM.docx]

**Supplementary Material**

**Table S1-a.** Characteristics of lean subjects and obese subjects before and after weight loss (WL) or control (CRTL) interventions.

|  | Lean | WL(D1) | WL(D2) | CTRL(D1) | CTRL(D2) |
| --- | --- | --- | --- | --- | --- |
| Number | 15 | 14 | 14 | 15 | 15 |
| Age(y) | 47.4 ± 4.5 | 44 ± 3.7 |  | 44.8 ± 3.4 |  |
| BMI(kg/m^2^) | 23.0 ± 0.6 | 30.0 ± 0.5 | 26.9 ± 0.5* | 30.7 ± 0.7 | 30.4 ± 0.7 |
| Glucose (mol/L) | 5.1 ± 0.07 | 5.3 ± 0.12 | 5.0 ± 0.09 | 5.3 ± 0.09 | 5.3 ± 0.37 |
| HOMA(IR) | 1.62±0.1 | 3.00±0.4 | 1.75±0.2* | 2.89±0.4 | 2.92±0.3 |

Data are presented as mean ± SEMs. *: A significant effect of weight loss (*P*<0.05). WL: weight loss, CTRL: control, D1, D2: before and after intervention (see also Figure 1), BMI: Body mass index.

**Table S1-b.** Characteristics of the subjects of the polyphenol (PP) study before and after intervention.

|  | Placebo | | Polyphenol | |  |
| --- | --- | --- | --- | --- | --- |
|  | Week 0 | Week 12 | Week 0 | Week 12 | |
| Number | 14 |  | 13 |  | |
| Age (y) | 41.4 ± 2.5 |  | 35 ± 3.1 |  | |
| BMI (kg/m^2^) | 28.5 ± 0.8 |  | 29.6 ± 0.8 |  | |
| Glucose(mol/L) | 5.1 ± 0.1 | 5.1 ± 0.1 | 5.1 ± 0.07 | 5.1 ± 0.1 | |
| HOMA(IR) | 2.2± 0.4 | 2.3 ± 0.3 | 1.9 ± 0.2 | 1.7 ± 0.1 | |
| RQ | 0.78 ± 0.01 | 0.82 ± 0.02 | 0.80 ± 0.01 | 0.79 ± 0.01 | |
| ΔRQ | 0.08 ± 0.01 | 0.06 ± 0.01 | 0.07 ± 0.01 | 0.08 ± 0.01 | |
| Fat oxidation | 0.08 ± 0.006 | 0.06 ± 0.01 | 0.07 ± 0.005 | 0.07 ± 0.008 | |

Data are means ± SEMs. BMI: Body mass index

**Table S2A.** Univariate comparisons of effect of weight loss intervention on baseline values in obese subjects (ΔWLT0 vs. Δ CTRL T0).

| Metabolite | P | lFDR |
| --- | --- | --- |
| *Amino acids & related metabolites* |  |  |
| Creatinine | <0.01 | NS |
| Glycine | <0.01 | NS |
| 2 aminoadipic acid | 0.01 | NS |
| Isoleucine | 0.01 | NS |
| Leucine | 0.01 | NS |
| Creatine | 0.02 | NS |
| Carnitine | 0.02 | NS |
| Betaine | 0.02 | NS |
| 4 hydroxyproline | 0.03 | NS |
| Tyrosine | 0.05 | NS |
| Valine | 0.05 | NS |
| *Acylcarnitines* |  |  |
| Propionylcarnitine (C3) | <0.01 | NS |
| 2-Methylbutyroylcarnitine (C5) | <0.01 | NS |
| *TCA cycle & related metabolites* |  |  |
| Succinic acid | 0.03 | NS |
| Glutamic acid | 0.04 | NS |

*P*<0.05 was considered significant. lFDR: local false discovery rate

**Table S2B.** Comparison of effect of weight loss intervention on challenge response values in

obese subjects (iAUC WL vs. iAUC CTRL).

| Metabolites | *Day*Condition* | lFDR |
| --- | --- | --- |
| *Amino acids & related metabolites* |  |  |
| SDMA | 0.02 | NS |
| Dimethylglycine | 0.03 | NS |
| Methionine | 0.04 | NS |
| Asparagine | 0.04 | NS |
| Succinate | 0.04 | NS |
| Arginine | 0.05 | NS |
| *Acylcarnitines* |  |  |
| Myristoyl (C12) | 0.05 | NS |
| Choline | 0.05 | NS |
| *Oxylipins* |  |  |
| 12.13.DiHOME | <0.01 | NS |
| 12S.HHTrE | 0.04 | NS |
| 9.10.DiHOME | 0.04 | NS |
|  |  |  |

*P*<0.05 was considered significant subjects according to

univariate linear mixed models. Day*Condition : Interaction

between Day and Condition ,lFDR: local false discovery rate

**Table S3A.**Univariate comparisons of effect of polyphenol intervention on baseline values in

obese subjects (Δ Polyphenol T0 vs. Δ Placebo T0)

| Metabolite | P | lFDR |
| --- | --- | --- |
| *Amino acids & related metabolites* |  |  |
| Histidine | 0.03 | NS |
| Dopamine | 0.05 | NS |
| *Acylcarnitines* |  |  |
| Octanoyl (C8) | 0.01 | NS |
| Decanoyl (C10) | 0.03 | NS |

*P*<0.05 was considered significant. lFDR: local false discovery rate

**Table S3B.** Comparison of effect of the polyphenol intervention on challenge response values in

obese subjects (iAUC Polyphenol vs. iAUC Placebo).

| Metabolite | Day*Group | lFDR |
| --- | --- | --- |
| *Amino acids & related metabolites* |  |  |
| Cystathionine | 0.01 | NS |
| *Acylcarnitines* |  |  |
| Myristoyl (C12) | 0.01 | NS |
| Deoxy (C0) | 0.01 | NS |
| Isovaleryl (C5) | 0.05 | NS |
| *Organic acids* |  |  |
| 3 Hydroxybutyric acid | 0.04 | NS |

*P*<0.05 was considered significant. lFDR: local false discovery rate

**Table S4.** Univariate correlation of the fasting plasma metabolome of volunteers in polyphenol intervention study with HOMA, fat oxidation and ΔRQ.

|  | ∆RQ | | HOMA | | FAT Oxidation | |
| --- | --- | --- | --- | --- | --- | --- |
| Metabolites | P | ρ | P | ρ | P | ρ |
| Proline | <0.01 | -0.42 |  |  |  |  |
| Tiglylcarnitine | <0.01 | -0.39 |  |  |  |  |
| Isovalerylcarnitine | <0.01 | -0.38 |  |  |  |  |
| Glutathione | 0.01 | 0.34 | 0.03 | -0.30 |  |  |
| Betaine | 0.01 | -0.33 |  |  |  |  |
| Glycylglycine | 0.02 | 0.32 |  |  |  |  |
| Methionine.sulfone | 0.03 | 0.30 |  |  |  |  |
| Saccharopine | 0.03 | -0.30 |  |  |  |  |
| Pivaloylcarnitine | 0.04 | -0.30 |  |  |  |  |
| DL-3.aminoisobutyric acid | 0.04 | -0.28 |  |  |  |  |
| Propionylcarnitine | 0.05 | -0.28 |  |  |  |  |
| 3.Methoxytyramine |  |  | <0.01 | 0.48 |  |  |
| Citric acid |  |  | <0.01 | -0.46 |  |  |
| Beta Alanine |  |  | <0.01 | 0.41 |  |  |
| Acetylcarnitine |  |  | <0.01 | -0.37 |  |  |
| Oleylcarnitine |  |  | 0.01 | -0.36 |  |  |
| Asparagine |  |  | 0.02 | -0.33 |  |  |
| 4.hydroxyproline |  |  | 0.02 | 0.32 |  |  |
| Norepinephrine |  |  | 0.03 | -0.32 |  |  |
| Tyrosine |  |  | 0.03 | 0.31 |  |  |
| Cis-aconitic acid |  |  | 0.03 | -0.30 |  |  |
| Homocysteine |  |  |  |  | <0.01 | 0.40 |
| S-methylcysteine |  |  |  |  | <0.01 | 0.40 |
| Kynurenine |  |  |  |  | 0.01 | 0.36 |
| Succinic acid |  |  |  |  | 0.01 | -0.34 |
| Cysteine |  |  |  |  | 0.02 | 0.32 |
| Cystathionine |  |  |  |  | 0.04 | 0.28 |

ρ: Pearson correlation coefficient.

**Table S5.** Goeman’s global testing of differences in metabolic pathways observed during the postprandial response, expressed as piAUC. Goeman’s global tests were performed separately for volunteers from the weight loss and polyphenol intervention studies. No significant effects were observed for effect of weight loss or polyphenol intervention on postprandial response, expressed as piAUC (data not shown).

| Pathways and number of metabolites |  | Difference  obese vs lean | Correlation with HOMA for weight loss intervention group |
| --- | --- | --- | --- |
| TCA cycle | 8 | NS | NS |
| BCAA catabolism | 7 | NS | NS |
| Fatty acid oxidation | 17 | NS | NS |
| Lactate pathway | 3 | 0.06 | 0.05 |
| Arg, Pro | 6 | 0.1 | NS |
| Phe, Tyr | 2 | NS | NS |
| Enzymatic oxidation of arachidonic acid | 5 | NS | NS |
