# Supplementary material for: Global testing of shifts in metabolic phenotype
Source: Metabolomics. 2018 Oct 4;14(10):139. doi: 10.1007/s11306-018-1435-8 (PMC6208751; doi:10.1007/s11306-018-1435-8)
Supplement: Supplementary file 2 — Supplementary material 2 (DOCX 13 KB) [file 11306_2018_1435_MOESM2_ESM.docx]

**Table S6.** Amino acid (AAAC) and fatty acid (FAAC) derived acylcarnitines covered in this study.

|  | -Carnitine | Abbreviation | Origin |
| --- | --- | --- | --- |
| AAAC (C_2-5_) | Acetyl- | C_2_ | Leu, Ile |
|  | Proprionyl- | C_3_ | Ile, Val |
|  | Isobutyryl- | C_4_ | Val |
|  | 2-Methylbutyryl- | C_5-Ile_ | Ile |
|  | Isovaleryl- | C_5-Leu_ | Leu |
| FAAC (C_6-18_) | Hexanoyl- | C_6:0_ | Caproic, hexanoic acid |
|  | Octanoyl- | C_8:0_ | Octanoic acid |
|  | Octenoyl- | C_8:1_ | Octenoic acid |
|  | Nonayl | C_9.0_ |  |
|  | Decanoyl- | C_10:0_ | Decanoiccapric acid |
|  | Decenoyl- | C_10.1_ |  |
|  | Lauroyl- | C_12:0_ | Lauric acid |
|  | Dodecenoyl- | C_12:1_ |  |
|  | Myristoyl- | C_14:0_ | Myristic acid |
|  | Tetradecenoyl- | C_14:1_ |  |
|  | Tetradecadienyl | C_14:2_ |  |
|  | Palmitoyl- | C_16:0_ | Palmitic acid |
|  | Hexadecenoyl | C_16:0_ |  |
|  | Stearoyl- | C_18:0_ | Stearic acid |
|  | Oleyl | C_18:1_ | Oleic acid |
|  | Linoleyl- | C_18:2_ | Linoleic acid |
